# Phytochemical composition, and cytotoxic, antioxidant, and antibacterial activity of the essential oil and methanol extract of *Semenovia suffruticosa*

**Published:** 2019

**Authors:** Sara Soltanian, Neda Mohamadi, Peyman Rajaei, Mojtaba Khodami, Mehdi Mohammadi

**Affiliations:** 1 *Department of Biology, Faculty of Science, Shahid Bahonar University of Kerman, Kerman, Iran.*; 2 *Student Research Committee, Kerman University of Medical Sciences, Kerman, Iran.*; 3 *Pharmaceutics Research Center, Institute of Neuropharmacology, Kerman University of Medical Sciences, Kerman, Iran.*; 4 *Department of Biology, Kerman Branch, Islamic Azad University, Kerman, Iran.*; 5 *Neuroscience Research Center, Kerman University of Medical Science, Kerman, Iran.*

**Keywords:** Semenovia suffruticosa, Essential oil, Methanol extract, Antioxidant and cytotoxic properties, Antibacterial activity

## Abstract

**Objective:**

In this study, our aim was to extract, and identify and quantify the chemical composition of essential oils of *Semenovia suffruticosa* grown in Kerman, Iran. Moreover, cytotoxic, antioxidant and antimicrobial activity of the essential oil and methanol extract of aerial parts of *S. suffruticosa* were reported.

**Materials and Methods:**

GC and GC/MS analysis were used for identifying and quantifying the essential oil components. Antioxidant and antibacterial activity were tested by 2, 2-diphenyl-1-picrylhydrazyl (DPPH) and agar disc diffusion methods, respectively and MTT assay was used to determine the anti-proliferative potential of the oil against breast (MCF-7), colon (HT-29), neuroblastoma (SH-SY5Y), embryonal carcinoma (NCCIT) cancer cell relative to human umbilical vein endothelial cell (HUVEC) as a normal cell. Apoptosis induction was monitored by ﬂow cytometry using PE annexin V apoptosis detection kit and cell cycle arrest was by with propidium iodide.

**Results:**

Z-β-ocimene (25.1%), linalool (17.8%) and β-bisabolol (13.3%) were recognized as major components of the essential oil. Our study demonstrated apoptosis-inducing potential of essential oil on normal and cancer cells. However, methanol extract exerted cytotoxicity against a number of cancer cells and arrested cancer cells in G2/M phase; nevertheless, it did not exert strong cytotoxicity against normal cells. Furthermore, DPPH and disc diffusion results showed that while essential oil has considerable antiradical activity, methanol extract did not exert promising antioxidant and antimicrobial activity.

**Conclusion:**

Methanol extract of *S. suffruticosa* shows tumor-cell-specific cytotoxic properties and the essential oil demonstrated a strong antioxidant activity.

## Introduction

Nowadays, many researches are conducted for finding natural components which possess a variety of biological activities such as antioxidant, antibacterial and anti-cancer potentials (Hammami et al., 2015 ▶; Losso et al., 2007 ▶; Scalbert et al., 2005 ▶). Generally, plants are rich sources of antioxidant components that can reduce the effect of oxidative damage caused by free radicals and protect the body against oxidative-related diseases such as cancer (Christaki et al., 2012 ▶; Shukla et al., 2016 ▶; Lin et al., 2015 ▶; Hasani-Ranjbar et al., 2009 ▶; Nirmala et al., 2011 ▶; Morteza-Semnani, 2015 ▶). Moreover, many of plant-derived natural products have anti-proliferative/cytotoxic effects on cancer cells and in comparison to conventional chemical drugs, have fewer side effects (Wu et al., 2005 ▶). In addition, synthetic antibiotics which have resulted in the emergence of resistant microorganisms, are going to be replaced by natural products with antimicrobial effects (Lai et al., 2004 ▶; Bamoniri et al., 2010 ▶; Sahraei et al., 2014 ▶). In this regard, discovering endemic medicinal plants in each country and their pharmacologically bioactive compounds has attracted researchers. 

The genus *Semenovia* belongs to Apiaceae family and consists of twenty seven species. Eleven species are found in Iran and five of them including *S. tragioides*, *S. frigida, S. suffruticosa, S. subscaposa*, and *S. dichotoma *are endemic species to Iran (Rechinger and Teucrium, 1987 ▶; Rechinger, 1987 ▶). *Semenovia suffruticosa* (Freyn et Bornm. Manden.) is one of these endemic plants which grow in Kerman, Yazd and Zahedan provinces. In previous studies, the composition of the essential oil of *S. suffruticosa *from Taftan mountain, Zahedan and from Shirkouh mountain, Yazd, Iran (Mottaghipisheh et al., 2017 ▶; Rustaiyan et al., 1999 ▶) were reported. The aim of this study was to identify the chemical composition of the essential oil of *S. suffruticosa *collected from Lalehzar mountain, Kerman, Iran. Furthermore, some biological activities such as antioxidant, antimicrobial and anti-cancer properties of the essential oil and methanol extract of *S. suffruticosa *were reported for the first time.

## Materials and Methods

**Plant material**

The aerial parts of the plant were harvested in summer (2016) from Lalehzar Mountain (40R05435550, UTM3327896) in Kerman Province, at 3200 m altitude. The plant was dried in shade and a voucher specimen was deposited in Islamic Azad University, Kerman branch, Iran. 

**Preparation of the methanol extract**

Dried *S. suffruticosa *aerial parts were powdered and sieved using a 300-mesh sieve. The samples (100 g) were placed in a flask and macerated using 400 ml warm water at room temperature (28°C) for 72 hr with occasional stirring. *S. suffruticosa *samples (50 g) were mixed with 500 ml methanol (98% v/v. Merck, Germany). The mixture was placed in an ultrasonic bath at 40°C and continuously sonicated for 50 min. Extraction was repeated until it became clear. The suspension was filtered through a filter paper to remove solid particles. The residue was placed in an oven (38°C) to dry. The crude extract was stored at -20°C.

**Isolation of essential oil**

Here, 50 g of dried aerial parts of the plant was subjected to hydro distillation for 2 hr using Clevenger-type apparatus to yield 0.5% essential oil.

**Gas Chromatography (GC) and Gas chromatography/mass spectrometry (GC/MS) analysis**

GC-MS analysis was done using a Hewlett-Packard 5973 mass spectrometer coupled with a Hewlett-Packard 6890 gas chromatography equipped with a HP-5MS capillary column (5% phenyl methylpolysiloxane, 30m×0.25mm, film thickness 0.25 μm). The carrier gas was helium. All mass spectra were acquired in electron-impact (EI) mode with an ionization voltage of 70 eV. For identification of essential oil components, relative retention time and mass spectra of each compound were compared with the standards. The components of essential oil were identified by comparison of their mass spectra and retention indices relative to n-alkanes of $C_{8}$-$C_{32}$ with data in the NISt and Wiley library (Adams, 2001 ▶).

**DPPH free radical scavenging assay**

Antioxidant potential of the plant essential oil and extracts was examined using 2, 2-diphenyl-1-picrylhydrazyl (DPPH) scavenging assay. Here, 50µl of different concentrations of each sample was added to 150 μl of methanol solution of 0.004% DPPH and incubated at room temperature for 30min; then, absorbance was read at 517nm (Burits and Bucar, 2000 ▶). Butylated hydroxytoluene (BHT) was used as reference drug. The percentage of inhibition was calculated as follow: I%=(A_con_-A_sam_)/A_sam_×100, where A_con _and A_sam_ are the absorbance of control and sample, respectively. IC50 values of the extract and oil (i.e. concentration that is necessary to decrease the initial concentration of DPPH by 50%) were calculated.

**Cell lines and culture medium**

Human breast (MCF-7), colon (HT-29), neuroblastoma (SH-SY5Y), embryonal carcinoma (NCCIT) cancer cell lines and human umbilical vein endothelial cell (HUVEC) as a normal cell line, were used during the experiments. Cell lines were purchased from the Cell Bank of Pasteur Institute, Tehran, Iran. MCF-7, HT-29, SH-SY5Y and HUVEC were maintained in Dulbecco’s Modifed Eagle’s Medium (DMEM; Gibco) and NCCIT were cultured in RPMI 1640 (Gibco). Both medium was supplemented with 10% of fetal bovine serum (FBS) and 1% of penicillin and streptomycin. Cells were grown at 37°C in a humidified atmosphere with 5% CO_2_. 

**Preparation of samples**

In order to prepare different concentrations of the extract, first, 500 mg of the plant extract was dissolved in 1ml dimethyl sulfoxide (DMSO, Merck), and diluted with complete culture medium to have 10 mg/ml stock solution. Finally, the desired concentration of extract was obtained by diluting 10 mg/ml stock with complete culture medium. The essential oil was dissolved in 0.5% DMSO in complete culture medium and then, intended dilutions (0.026, 0.08, 0.24, 0.72, 0.21, 6.4, and 19.4 µl/ml) were prepared for MTT assay. 

**Cytotoxic activity**

The cytotoxicity of the methanol extracts and essential oil of *S. suffruticosa* on different cell lines including MCF-7, HT29, NCCIT, SH-SY5Y and HUVEC were measured using 3- (4, 5-dimethylthiazol-2-yl)-2, 5-diphenyl tetrazolium bromide (MTT) assay. The cells were grown in 96-well plates. After 24 hr, the culture medium was removed and cells were treated with medium containing increasing concentrations of methanol extract and essential oil in separate experiments and incubated for 48 hr. Later, 20 µl of the MTT solution (5 mg/ml) was added to each well and the plate was re-incubated for 3 hr. Finally, the medium was removed and 100 µl of DMSO was added to dissolve formazan crystals. The amount of formazan crystal was determined by measuring the absorbance at 490 nm by using a multi-plate reader (ELISA reader; BioTek-ELx800, USA). Since the solvent (DMSO) has cytotoxic effects, the control treatments were treated with equivalent amount of DMSO. The experiments were performed in triplicate and the ratio of the absorbance of treated cells to the absorbance of DMSO-treated control cells was regarded as cell viability (%). The concentration providing 50% inhibition (IC50) was calculated from a graph plotting cell viability percentage against different methanol extract or essential oil concentrations.

**Apoptosis assay by flow cytometry**

PE Annexin V versus 7-aminoactinomycin D (7-AAD) staining was performed according to the manufacturer’s instructions using PE Annexin V Apoptosis Detection Kit I (BD Biosciences) and analyzed by ﬂow cytometry. Briefly, cells were plated into 6-well culture dishes (3×10^5 ^cells/well) for 24 hr prior to the addition of the essential oil (5 µl/mL). Following 24-hr incubation with the essential oil, the percentage of apoptotic cells was determined by the annexin V-PE/7-AAD assay. The cells were harvested, washed with cold phosphate-buffered saline and suspended in 1X binding buffer at a concentration of 1×10^6^ cells/ml. Then, 100µl of the cell suspension was added to a tube, treated with 5 µl of PE Annexin V and 5 µl of 7-AAD and incubated for 15 min at room temperature in the dark. Finally, the fluorescence of the cells was immediately determined by a flow cytometer (Partec FloMax, Münster, Germany) using fl2 and fl3 filters for detection of PE-Annexin and 7-AAD.

**Cell cycle analysis**

Flow cytometry was performed to identify the proportion of cells in each of the three interphase stages of the cell cycle. Approximately, 2.5×10^5^ MCF-7 cells were seeded into a 24-well plates. Cells were then treated with *S. suffruticosa *methanol extract (320 µg/mL) for 48 hr. The cells were detached from the culture dish using trypsin/EDTA, washed twice with ice-cold PBS and fixed with 70% ice-cold ethanol for 30 min. After washing, cells were re-suspended in 1 ml PBS containing 20 µg/mL propidium iodide and 10 μg/mL RNase A and incubated for 1 hr at 37°C in the dark. DNA content and cell cycle progression were studied by ﬂow cytometry using FACSCalibur^™ ^(Becton Dickinson, USA) and results were analyzed using Cell Quest^TM^ Pro software. 

**Disc diffusion assay**

The methanol extract of *S. suffruticosa *was evaluated for antibacterial activity against some pathogenic bacteria by agar disc diffusion method (NCCLS, 1997). The plant extract was dissolved in deionized water to a final concentration of 1000 mg/ml and filtered through 0.45 µm Millipore filters for sterilization. 

100 µl of bacterial suspension was poured on each plate containing Muller-Hinton Agar (MHA, Merck) to achieve a concentration of 1-2×10^8^ CFU/mL

The discs (6 mm in diameter) impregnated with different concentrations (10, 20, 25, 30, 50 and 100 µg/mL) of the extract solution and deionized water (as negative control) were placed on the inoculated agar. The inoculated plates were incubated for 24 hr at 37^o^C. Tetracycline (30µg/mL) was used as positive control. The diameters of inhibition zones were considered a measure of antimicrobial activity and each assay was repeated twice.

**Microbial strains**

Methanol extract of *S. suffruticosa *was individually tested against a panel of 8 microorganisms. Microbial strains used in this research were *Pseudomonas aeruginosa* (ATCC 27853), *Escherichia coli* (ATCC 10536), *Bacillus subtilis* (ATCC 6633), *Klebsiella pneumoniae* (ATCC 10031), *Acinetobacter baumannii* (ATCC 19606), *Enterococcus faecalis* (ATCC 29212), *Bacillus cereus* (ATCC 21366), and *Micrococcus luteus* (MTCC 2470). Bacterial strains were cultured for 24 hr at 37^o^C in Muller-Hinton Agar (MHA, Merck).

**Statistical analysis**

Data are expressed as mean±SD of three experiments. Statistical analyses were performed using SPSS version 16. Statistical significance was determined using Student’s t-test for comparisons between treated versus control cells and a p<0.05 was considered statistically significant

## Results

**Chemical Composition of the Essential Oil**

The aerial parts of *S. suffruticosa *were subjected to steam distillation using the Clevenger-type apparatus to yield 0.3% v/w of yellowish oil. In total, 33 compounds were identified, representing 92% of the total oil components (Table 1). The major components of the oil were Z-β-ocimene, linalool, β-bisabolol representing 56.2% of the total composition. 

**Table 1:** Chemical composition of the essential oil of the leaves and flowers of *S. suffruticosa*

**No**	**Compound**	**Retention Indices**	**Percentage**
**1**	α-Pinene	939	0.1
**2**	Camphene	954	0.3
**3**	Sabinene	975	4.4
**4**	β-Pinene	979	1.8
**5**	Myrcene	991	0.9
**6**	α-Terpinene	1017	0.1
**7**	*p-*Cymene	1025	1.9
**8**	Limonene	1029	0.9
**9**	1,8-Cineole	1031	1.0
**10**	Z-β-Ocimene	1037	25.1
**11**	E-β-Ocimene	1050	8.4
**12**	γ-Terpinene	1060	1.7
**13**	*cis*-Sabinene hydrate	1070	0.1
**14**	*p*-Cresol	1076	0.5
**15**	α-Terpinolene	1089	0.2
**16**	Linalool	1097	17.8
**17**	Isopentyl-2-methyl butanoate	1100	1.1
**18**	allo-Ocimene	1132	0.4
**19**	Terpinene-4-ol	1177	0.4
**20**	α-Terpineol	1189	0.6
**21**	Methyl Chavicol	1196	-
**22**	Piperitone	1253	-
**23**	Bornyl acetate	1289	-
**24**	Lavandulyl acetate	1299	3.7
**25**	*cis*-Jasmone	1393	0.3
**26**	Methyl Eugenol	1404	0.1
**27**	*trans-*Caryophyllene	1419	1.5
**28**	Germacrene-D	1455	0.1
**29**	BicycloGermacrene	1500	1.7
**30**	δ-Cadinene	1523	0.1
**31**	Spathulenol	1578	4.3
**32**	Caryophyllene oxide	1583	0.1
**33**	β-Bisabolol	1686	13.3
	total		92.9

**DPPH radical scavenging activity**

The DPPH scavenging activity of the methanol extract and essential oil was measured by the bleaching of the purple methanol solution of DPPH. The results of DPPH radical scavenging activity of BHT (standard), methanol extract and essential oil of *S. suffroticosa* are shown in Table 2. 

**Table 2:** The inhibitory concentration 50% (IC50) of methanol extract and essential oils of *S. suffroticosa* in DPPH test (Mean±SD)

	**Methanol extract (µg/mL)**	**Essential oil (µL/mL)**	**BHT (µg/mL)**	**BHT (µL/mL)**
**IC50**	693.3±33.5	0.42±0.01	7.45±1.08	2.42±0.12

**Cytotoxic activity**

**Figure 1:**
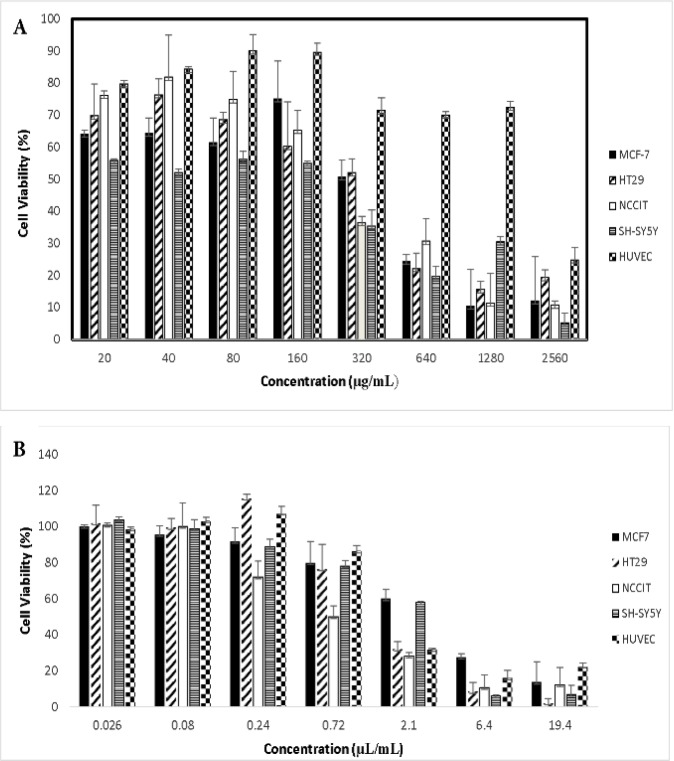
Cytotoxic activity of the methanol extract (A) and essential oil (B) of *S. suffruticosa *on cancerous and normal cell lines. MCF-7, HT-29, NCCIT, SH-SY5Y and HUVEC Cell lines were cultured and treated with various concentrations of the extract (20-2560 µg/mL) and oil (0.026-19.4 µl/ml) for 48 hr and cell viability percentage was measured. All data are reported as mean ±SD of at least three separate experiments

In order to study the possible anti-proliferative activity of methanol extract and essential oil of *S. suffruticosa*, *in vitro* cytotoxic analysis using MTT colorimetric assay on different tumor cells and one normal cell lines was performed. A relatively dose-dependent inhibitory effect on the proliferation of all tested cancer cell lines was detected for this extract. Although, growth inhibitory effect of the extract on NCCIT (IC50; 242 µg/mL) and SH-SY5Y (IC50; 220 µg/mL) was stronger than that against MCF-7 (IC50; 320 µg/mL) and HT29 (IC50; 341 µg/mL), but there was not much difference among susceptibility of different cancer cell lines to the plant extract. However, this study demonstrated that this extract displays low inhibitory effect on proliferation of normal HUVEV cell line with an IC50 of 1866 µg/mL after 48-hr incubation (Figure 1A). In order to test cytotoxic effects, cells were exposed to 0.026, 0.08, 0.24, 0.72, 2.1, 6.4 and 19.4 µL/mL of the essential oil for 48 hr. Our findings indicated essential oil-induced cell death in a dose-dependent manner in all cancer cell lines (MCF-7, HT29, SH-SY5Y and NCCIT) as well as HUVEC as a normal cell line (Figure 1B). A microscopic examination further revealed morphological changes in all cell lines following exposure to the essential oil which resulted in suspension of dead cells in the medium (data not shown). The IC50 values of the essential oil at 48 hr were 5, 4.2, 1.6, 1.5 and 0.72 µL/mL against MFC-7, SH-SY5Y, HUVEC, HT29 and NCCIT, respectively. 

**Essential oil induced apoptosis of MCF-7**

**Figure 2:**
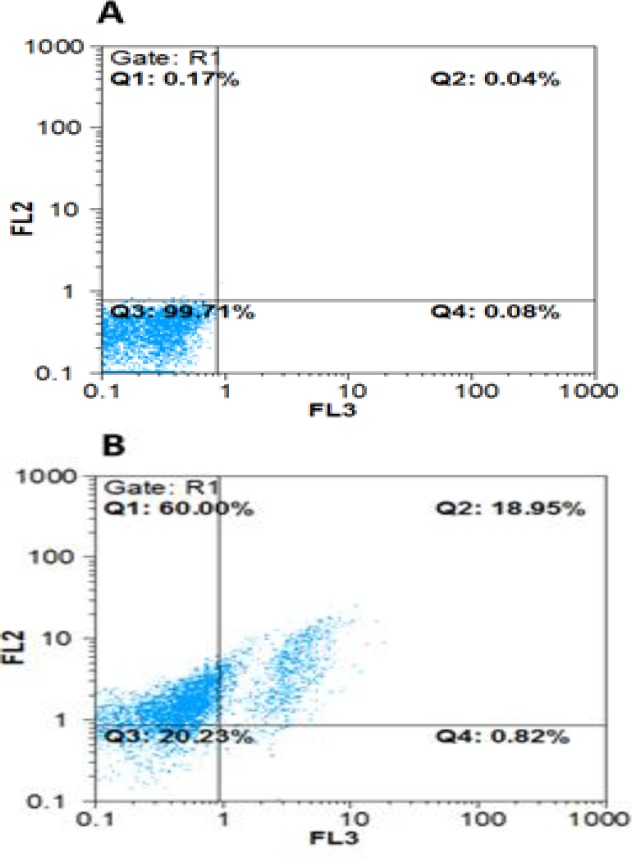
Apoptotic effect of *S. suffruticosa *essential oil on MCF-7 cell line was assessed using PE Annexin V Apoptosis Detection Kit I. Untreated cells (A), and cells treated with 5µl/ml of essential oil for 48 hr (B) were analyzed using flow cytometer. PE Annexin V and 7-AAD negative (Q3) were considered viable cells; live-gated cells within the Annexin-V^+^7AAD^-^ compartment (Q1) were identified as early apoptotic cells and gated cells within the Annexin-V^+^7AAD^+^ compartment (Q2) were identified as late apoptotic and gated cells with the Annexin V^-^ 7AAD^+^ (Q4) were considered necrotic cells

7-AAD-annexin-V double staining was used to differentiate intact cells from early apoptotic, late apoptotic, and dead (necrotic) cells. FACS analysis on the control untreated cells revealed that the untreated cells were primarily annexinV-FITC and propidium iodide negative indicating that they were viable and not undergoing apoptosis (Figure 2A). After incubation of MCF-7 cells with the essential oil at 5µl/ml for 48 hr, the majority of cells exhibited an early apoptotic phenotype (60%) but 18% of cells were in the late apoptosis/necrotic phase (Figure 2B). 

**Methanol extract-induced cell cycle arrest in MCF-7 cell line**

**Figure 3:**
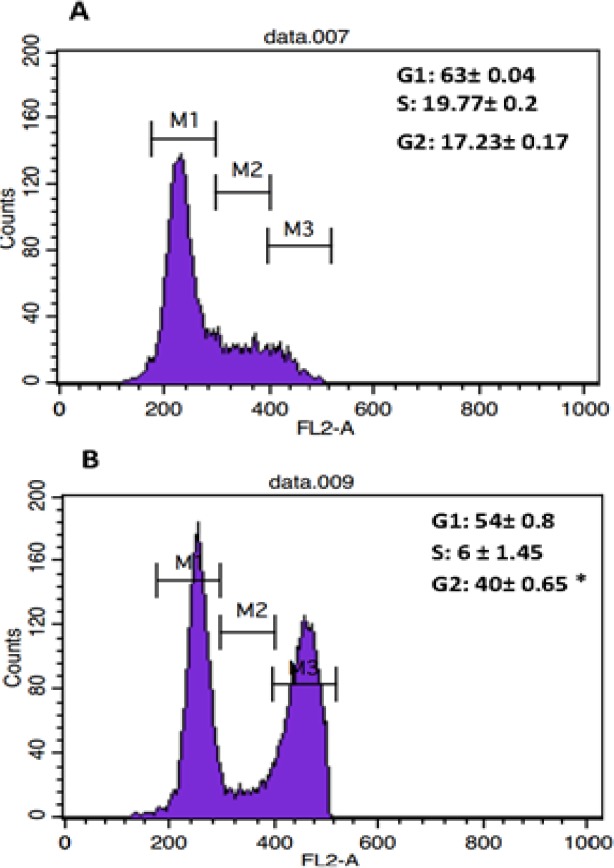
The effect of *S. suffruticosa *extract on cell cycle progression in MCF-7 cells. Untreated cells (A) and MCF-7 treated with *S. suffruticosa *extract (320 μg/ml) for 48 hr (B) were stained with PI and analyzed using BD FACScan ﬂow cytometer. The DNA histogram shows the distribution and percentage of cells in different phases of cell cycle. Results are expressed as mean±SD of 3 independent experiments. *p<0.05 shows significant differences as compared to the control as tested by the Student’s t-test. Each DNA histogram represents one of the three independent experiments

To investigate if the inhibitory effect of the extracts on the cell lines was due to induction of cell cycle arrest, MCF-7 cell line was selected and treated with *S. suffruticosa *extract at a concentration equal to IC50 for 48 hr; flow cytometry was used to analyze cell cycle progression. Decrease in the S phase population was accompanied by significant increases in G2/M phase population (p<0.05) after 48-hr treatment compared to the control (Figure 3).

**Table 3:** Antimicrobial activity of the methanol extract of *S. suffruticosa*

	**Methanol extract**		**Antibiotic ** ***(*** **Tetracycline)**
**Test microorganisms**	DD	MIC	MIC/MBC
**Pseudomonas aeruginosa ATCC 27853**	-	-	-
**Escherichia coli ATCC 10536**	-	-	30 µg/ml
**Bacillus subtilis ATCC 6633**	-	-	30 µg/ml
**Klebsiella pneumoniae ATCC 10031**	-	-	30 µg/ml
**Acinetobacter baumannii ATCC 19606**	-	-	30 µg/ml
**Enterococcus faecalis ATCC 29212**	-	-	-
**Bacillus cereus ATCC 21366**	-	-	30 µg/ml
**Micrococcus luteus MTCC 2470**	-	-	30 µg/ml

A dash (-) indicates no antimicrobial activity.

DD: Inhibition zone in diameter (mm) around the impregnated discs.MIC: Minimal inhibition concentrations (as µg/ml).

MBC: minimum bactericidal *concentration *(as µg/ml).

**Antimicrobial activity**

The antimicrobial activity of methanol extract of *S. suffruticosa *against a panel of 8 microorganisms was assessed with respect to the presence or absence of inhibition zones and by calculation of zone diameters. Disc diffusion tests exhibited no significant antimicrobial activity for methanol extract against tested microorganisms (Table 3)

## Discussion

*S. suffruticosa *is a plant that is endemic to south-east of Iran (Kerman, Yazd and Zahedan provinces). Although in previous studies, essential oil compositions of this plant collected from Yazd and Zahedan provinces, were reported, there is no report on the biological activity of the plant’s essential oil and extract. 

Linalool (13.9%), lavandulyl acetate (11.5%), (E)-β-ocimene (8.6%), terpinen-4-o1(7.7%) and lavandulol (6.6%) were found to be the main constituents of the essential oil of *S. suffruticosa* collected from Shirkouh mountain, Yazd (Rustaiyan et al., 1999 ▶) and cis-β-ocimene (12.9%), linalool (9.5%), γ-terpinene (9.0%) and α-terpinolene (7.4%) were recognized as major components of *S. suffruticosa* collected from Taftan mountain, Zahedan (Mottaghipisheh et al., 2017 ▶). 

In this study, essential oil of *S. suffruticosa *collected from Lalehzar mountain in Kerman province was analyzed and Z-β-ocimene (25.1%), linalool (17.8%) and β-bisabolol (13.3%) were detected as main components. Ocimene and linalool are plant-derived monoterpene and β-bisabolol is a natural monocyclic sesquiterpene alcohol. Together, β-ocimene and linalool were identified among essential oil major compounds of *S. suffruticosa *collected from Yazd, Zahedan and Kerman, Iran. However, S.* suffruticosa *in each region has a particular composition of chemical compounds. Because the environmental factors can change the quantity and quality of the essential oil composition, it is possible to detect different components in the same plant such as *S. suffruticosa*, growing in different regions (Sardashti et al., 2012 ▶). Consequently, different biological activities could also be observed. 

Essential oil components analyzed in another species of *Semenovia* showed some similarity to *S. suffruticosa *(Ashraf and Bhatty, 1975 ▶; Masoudi Shiva et al., 2002 ▶; Masoudi Shiva et al., 2005 ▶; Masoudi Sh et al., 2011 ▶). 

However, there is no comprehensive report on the biological activity of *S. suffruticosa *essential oil and/or its extract. Our study fills this gap and examined antioxidant potential, and cytotoxic and antimicrobial effect of the essential oil and methanol extract of *S. suffruticosa. In* *vitro* radical scavenging and antioxidant capacity of the essential oil and methanol extract were studied by using DPPH free radical scavenging assay. The DPPH is a blue/purple stable radical that can undergo scavenging by antioxidant. Using DPPH assay, capacity of plant extracts and essential oils to donate hydrogen atom and/or electron to DPPH and converting it to a yellow molecule reflects its ability to scavenge free radicals (Lu and Foo, 2001 ▶; Da Porto et al., 2000 ▶; Tepe et al., 2005 ▶). BHT is a synthetic standard antioxidant that is used as positive control in evaluation of the anti-oxidative potential of various natural products. Compared to synthetic standard antioxidant BHT, essential oil of *S. suffruticosa* displayed very high radical-scavenging activity. Oxygenated monoterpenes and monoterpene hydrocarbons are the most abundant compounds in essential oil. These components that possess different functional groups (alcohols, aldehydes, ketones, ethers, etc.) have antioxidant properties and are responsible for high antioxidant potential of essential oil.

On the contrary, reducing power activity of the methanol extract was week. Due to the major role of phenolic compounds in antioxidant activity, low antioxidant activity of the plant extract may be a consequence of its low phenolic compounds content (Bamoniri et al., 2010 ▶). In another study, the results obtained by DPPH, FRAP and β-carotene/linoleic acid assays on antioxidant activity of leaf extracts of* S. suffruticosa* collected from Zahedan, Iran indicated ethanol and ethyl acetate fractions as the most powerful antioxidant of *S*. *suffruticosa *leaf extracts (Mottaghipisheh et al., 2017 ▶). 

Todays, natural plant compounds are used for curing many diseases such as diabetes, malaria, and oxidative damage-related diseases such as cancer. Lower toxicity of plant-derived components compared to that of chemo-drugs and their fewer adverse effects make them promising alternatives for therapeutic strategies (Cragg et al., 2009 ▶; Jung, 2014 ▶; Badmus et al., 2013 ▶). The present study evaluated cytotoxic potential of methanol extract and essential oil of *S. suffruticosa *against different cancer cell lines and one normal cell line. The results revealed that whereas, methanol extract exhibited considerable cytotoxicity in all tested cancer cell lines and low cytotoxic effect on normal cell, essential oil exerted high cytotoxicity against both normal and cancer cell lines. Therefore, methanol extract exerts tumor-specific activity and can be considered as a potential anti-cancer agent for furthers investigation. The cytotoxic effects of methanol extract and essential oil on MCF-7 cells were mediated through cell cycle arrest and inducing apoptosis. Exposure of phosphatidylserine on the external surface of the cell membrane which is recognized as a biomarker of apoptotic cells can be detected by annexin /7-AAD flow cytometric assays (Fadok et al., 1992 ▶). In this study, annexin/7-AAD assay confirmed the ability of the essential oil to induce early and late apoptosis. Cell cycle analysis also showed that methanol extract arrested MCF-7 cell cycle at the G2/M phase.

The results of disc diffusion test showed that methanol extract has no inhibitory activity against 8 microorganisms tested in this study. 

Ethyl acetate and chloroform extract of 

*S. suffruticosa *collected from Zahedan showed the highest antimicrobial activity against some gram-positive bacterial strains. Against gram-negative bacteria, polar and nonpolar extracts exhibited a mild inhibitory activity, in comparison with positive control (Mottaghipisheh et al., 2017 ▶; Masoudi Shiva et al., 2005 ▶; Masoudi Sh et al., 2011 ▶). Overall, *S. suffruticosa *extracts did not show significant antibacterial potentials. 

In conclusion, according to our study, essential oil constitutes of *S. suffruticosa *from Lalehzar Mountain, Kerman have some similar and some different components as compared to *S. suffruticosa *from Yazd and Zahedan province of Iran. In order to replace synthetic component by natural ones, evaluation of anti-cancer, antioxidant and antimicrobial activities of plant products is valuable. In this regard, our study is the first to report *in vitro* cytotoxic effect of the essential oil and methanol extract of *S. suffruticosa*. 

According to our findings methanol extract has potent anti-proliferative activities against cancer cell lines via induction of cell cycle arrest, whereas no cytotoxic effect was observed at higher dose on normal cells. Therefore, the extract contains bioactive components responsible for anti-cancer potential of plant extract; nonetheless, further studies are needed to isolate and characterize these components. Furthermore, significant antioxidant activity of the essential oil reflected by DPPH- test, encourages researchers to perform more detailed research in this respect. 
